# Deciphering the regulatory mechanism of neural behavioral decisions through biogenic amine-mediated modulation of neural circuits in *Caenorhabditis elegans*

**DOI:** 10.3389/fnsys.2026.1727180

**Published:** 2026-04-16

**Authors:** Hui Liu, Yunlong Shen, Dejian Peng

**Affiliations:** School of Basic Medicine, Yangtze University, Jingzhou, Hubei, China

**Keywords:** *Caenorhabditis elegans*, dopamine, neural circuit, octopamine, serotonin, tyramine

## Abstract

Brain function is not simply the sum of individual neuronal activities, but rather emerges from functional neural circuits comprising thousands to millions of neurons with specific topological structures and dynamic properties. Investigating the functions and information-processing architectures of these neural circuits is essential for understanding how the brain performs “computation” and “operation.” Biological neural regulatory networks are captivating due to their extensive utilization of diverse signaling molecules for interneuronal communication. Neurotransmitters, receptors, and neurons together compose neuroregulatory circuits, which act as fundamental functional units in the network for generating behavioral instructions. However, the discovery and decipherment of neural circuits remain challenging, due to the necessity of comprehending the functions of signaling molecules and neuronal cells, as well as their regulatory mechanisms. In this review, we focus on four biogenic amines signals, including dopamine, serotonin, octopamine and tyramine, and discuss their regulatory roles with their receptors in *Caenorhabditis elegans* neural circuits. In particular, we summarize the functional roles of the biogenic amine neurons and their complex interaction mechanisms in neural circuits. We also provide perspectives on the fine-scale neural connectivity, which will bridge microscopic neuronal activity with macroscopic cognitive behaviors, offering a theoretical framework for further elucidating the neural mechanisms underlying brain adaptation, learning, and memory.

## 1 Introduction

The brain performs a wide array of intricate functions, including the regulation of sensation, movement, emotion, and memory. Research on neural circuits spans multiple hierarchical levels, ranging from molecules and cells to the entire nervous system. Higher cognitive processes in humans, such as decision-making, consciousness, and social behavior, rely on the coordinated interplay of multiple brain circuits. Core questions in neuroscience include the nature of consciousness and its underlying neural mechanisms, as well as how neural circuits encode and generate behavioral outputs. Addressing these questions requires a comprehensive understanding of the anatomical organization of the nervous system, particularly the connectivity and functional coordination among different circuits. By elucidating the structural and functional relationships, we can not only tackle the fundamental scientific query of “how the brain functions,” but also provides theoretical and technical foundations for combating neurological disorders, and promoting human health and wellbeing.

Notably, neurological diseases such as Parkinson’s disease and epilepsy often arise from imbalances in specific neurotransmitter systems. Therefore, systematically analyzing the regulatory mechanisms of neurotransmitter networks and the dynamic characteristics of corresponding neural circuits not only provides new targets for modulating disease progression, but also establishes a theoretical foundation for precise drug design and neural modulation therapies.

Research on neural circuits in *Caenorhabditis elegans* (*C. elegans*) has become a major focus in neuroscience since White et al. (1986) provided a detailed description of the complete neuronal connections through anatomical experiments. Neural signaling in nematodes is a precise system, including neurons, neurotransmitters, neurotransmitter receptors, and the way of information regulation among them. Four biogenic amines, namely dopamine (DA), serotonin (5-HT), octopamine (OA), and tyramine (TA), have been identified as neurotransmitters for neuromodulation of various behaviors in *C. elegans* (Chase and Koelle, 2007). These biogenic amines are also known as monoamine neurotransmitters and only in a limited number of neurons (Horvitz et al., 1982; Sanyal et al., 2004). Neural circuits through these neurotransmitters integrate neural regulatory instructions such as excitation, inhibition, disexcitation (Liu et al., 2019) and disinhibition, to achieve amplification or attenuation of neural signals by expressing functional proteins or enzymes. This modulation enables animals to execute a broad range of behaviors, including sensation, movement, mating, social behavior, sleep, learning, and memory (de Bono and Bargmann, 1998; Barr and Garcia, 2006; Cronin et al., 2006; Raizen et al., 2008; Ardiel and Rankin, 2010).

Although nematodes lack some biogenic amines such as histamine and epinephrine that are present in vertebrates, the conserved roles of DA and 5-HT are evident across species, while OA and TA function similarly to epinephrine and norepinephrine in vertebrates (Bauknecht and Jekely, 2017). For instance, 5-HT levels rise during feeding (Sze et al., 2000; Voigt and Fink, 2015), and OA is released during starvation to enhance animal movements, as seen in *C. elegans* foraging (Dernovici et al., 2007), ant aggression (Aonuma and Watanabe, 2012), and fly hyperactivity (Selcho and Pauls, 2019). Moreover, *C. elegans* has developed a neural network modules capable of efficiently integrating information, analogous to those in mammals (Reigl et al., 2004; Sporns and Kotter, 2004). These findings underscore the value of nematode neural circuit research as a model for understanding general principles of nervous system function and behavior. In this review, we systematically examine the functional roles of biogenic amines and their neural circuit interaction mechanisms in the nervous system of *C. elegans*. We aim to integrate perspectives from neurobiology and cognitive science, offering a novel framework to address the fundamental question of how consciousness responds to the environment.

## 2 Neural circuits of DA and 5-HT

Both DA and 5-HT are synthesized by an amino acid decarboxylase BAS-1 in *C. elegans*. However, the rate-limiting enzyme for DA synthesis is tyrosine hydroxylase CAT-2, not BAS-1 (Hare and Loer, 2004). According to the expression pattern of CAT-2, DA is expressed in CEP, ADE, PDE, R5A, R7A, and R9A neurons. Among these, R5A, R7A, and R9A neurons are male-specific neurons in the male tail for mating (Barr and Garcia, 2006), the sensory neurons CEP, ADE, and PDE share related functional roles, including mechanosensation, movement coordination, foraging behavior as well as learning and memory (Sawin et al., 2000; Hills et al., 2004; Olivito et al., 2018; Cannizzaro et al., 2019; Salami et al., 2019).

**FIGURE 1:**
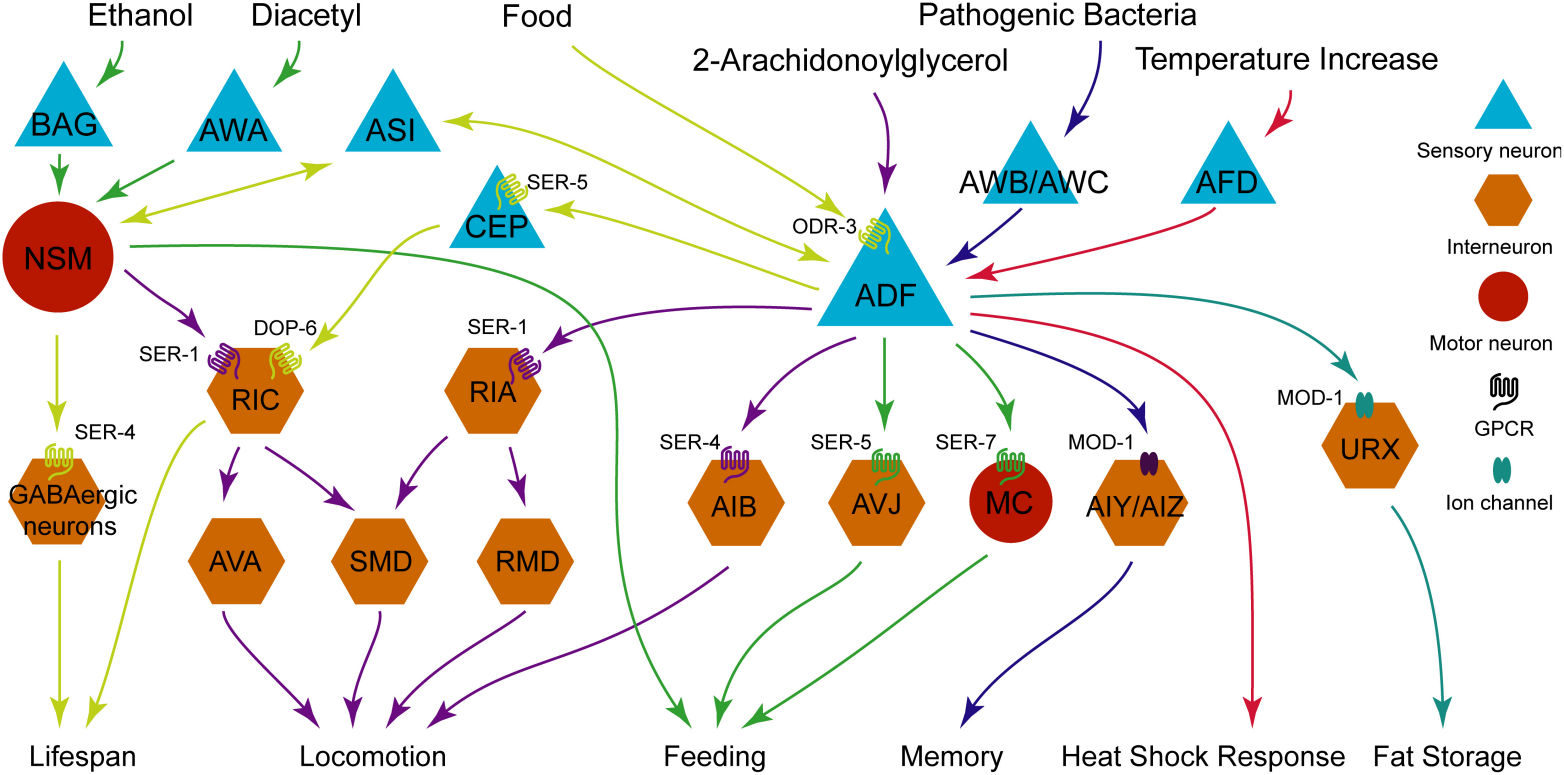
Diagram illustrating functional relationships between sensory neurons (blue triangles), interneurons (orange hexagons), motor neurons (red circles), with neuromodulators, GPCRs, and ion channels impacting processes like lifespan, locomotion, feeding, memory, heat shock response, and fat storage. Arrows indicate pathways and interaction effects.

The rate-limiting enzyme for 5-HT synthesis is tryptophan hydroxylase TPH-1 (Sze et al., 2000). Based on the expression pattern of TPH-1, 5-HT is synthesized in five neurons: ADF, NSM, HSN, AIM, and RIH. HSN neurons are located near the genital foramen of nematodes for egg-laying, and the function of AIM and RIH neurons function to recover 5-HT from the body (Sze et al., 2002). ADF and NSM neurons release 5-HT, which acts through G protein-coupled receptors (GPCR) SER-1, 4, 5, 7, and the chloride ion channel receptors MOD-1 in downstream neurons (Sze et al., 2002). Through these pathways, 5-HT regulates a wide range of physiological and behavioral processes, such as lifespan, foraging and movement, feeding, memory, heat shock response and fat storage (Dernovici et al., 2007; Cunningham et al., 2012; Noble et al., 2013; Qin et al., 2013; Song et al., 2013; Entchev et al., 2015; Lemieux et al., 2015; Tatum et al., 2015; Iwanir et al., 2016; Oakes et al., 2017; Williams et al., 2018; Mori et al., 2019; Singh and Aballay, 2020; Miller et al., 2022). This finding suggests the diversity in the connectivity and functions of 5-HT neural circuits. Analyzing 5-HT’s effects at a whole-brain scale is undoubtedly provide valuable insights, as it facilitates the identification of drug targets for neurological disorders (Dag et al., 2023). Therefore, synthesizing current knowledge of these fundamental circuits is a crucial step in scientific advancement. The circuits are described in detail below (Figure 1).

### 2.1 Lifespan

Lifespan of an organism is closely related to the quantity of available food. The lifespan of *C. elegans* varies with food concentration (from 3.5 × 10^10^ to 0 cells/mL): it initially increases, subsequently decreases, and then increases once more until reaching the plateau stage (below 10^7^ cells/mL), during which the lifespan is the longest (Entchev et al., 2015). Two genes, *tph-1* and *daf-7*, both participate in the regulation of the lifespan. In TPH-1 mutants (characterized by a lack of 5-HT synthesis), the lifespan is reduced under dietary restriction (DR) with a food concentration below 10^6^ cells/mL, whereas no significant difference under conditions of abundant food (Entchev et al., 2015). DAF-7 is a transforming growth factor beta (TGF-β) protein expressed only in ASI neurons (Nolan et al., 2002; Gumienny and Savage-Dunn, 2013). Starvation-induced expression decrease of DAF-7 or loss function of ASI neurons both prolong the lifespan (Bishop and Guarente, 2007). DAF-7 inhibits the expression of TPH-1 in NSM/ADF, and TPH-1 also inhibits the expression of DAF-7 in ASI. Therefore, the alteration of lifespan requires reciprocal regulation of 5-HT and TGF-β signaling (Entchev et al., 2015).

Both two serotoninergic neurons ADF and NSM play crucial roles in the regulation of lifespan. 5-HT released from NSM neurons binds two receptors SER-1 (Dernovici et al., 2007) and SER-4 (Tsalik et al., 2003). SER-1 exhibits relatively weak activity, while SER-4 is more effective and functions in GABA neurons. RIS neurons are the most probable GABA neurons but still require validation (Miller et al., 2022). In signaling pathway of ADF neurons, serotoninergic receptor SER-5 and dopaminergic DOP-6 function in CEP neurons and RIC neurons, respectively (Zhang et al., 2021). Therefore, ADF, CEP, and RIC neurons release 5-HT, DA, and OA, respectively, to regulate the DR-induced life extension (Figure 1, the part with yellow arrows).

Curiously, TPH-1 re-expression in NSM neurons, not in ADF neurons, rescued the defect of TPH-1 mutants (Miller et al., 2022). It seems that only NSM neurons regulate the DR-induced longevity, but blocking ADF neurons with tetanus toxin (TeTx) still abolished the lifespan suppression of food odor (Zhang et al., 2021). Furthermore in TPH-1 mutation, ADFs showed an obvious intracellular calcium flow in response to food odor, and olfactory receptor ODR-3 in ADF neurons was required for both ADF neurons’ calcium response and the odor inhibition of DR-induced longevity (Zhang et al., 2021). These findings demonstrate the function of ADF neurons in longevity regulation. In fact, besides 5-HT, ADF neurons also release glutamate and neuropeptides that exert their effects on downstream neurons (Nathoo et al., 2001; Kodama et al., 2006; Ohnishi et al., 2011; Chen et al., 2013). Therefore, there might be a synergistic effect involving one or several of these neurotransmitters.

### 2.2 Locomotion

The serotoninergic receptor SER-1, an ortholog of human serotoninergic receptor 2A (HTR2A) and HTR2B (Xiao et al., 2006), is required for feeding, egg-laying and locomotion (Chase and Koelle, 2007). According to the neuronal expression pattern of *ser-1p*:GFP (Green fluorescent protein), SER-1 is located in the head neurons RIA and RIC (Dernovici et al., 2007). In this pathway of locomotion regulation, RIA interneurons exhibit higher activity levels compared to RIC due to their direct synaptic connections with sensory neuron ADF and head motor neurons SMD and RMD (White et al., 1986). Furthermore, 5-HT receptors MOD-1 and SER-1 cooperate to regulate movement direction during foraging (Dernovici et al., 2007). MOD-1 is a serotoninergic receptor-gated chloride channel, which is also essential for locomotion regulation (Ranganathan et al., 2000). However, the cross-regulation of animal movement is very complicated (Figure 1, the part with purple arrows), the absence of MOD-1 in this neural circuit diagram is due to the fact that the specific neurons functionally expressing MOD-1 remain incompletely characterized.

Another GPCR SER-4, an ortholog of human HTR1A (Gurel et al., 2012), also inhibits animal forward movement (Carre-Pierrat et al., 2006). Both TPH-1 or SER-4 mutations downregulate the 2-arachidonoylglycerol (2-AG)-induced locomotory inhibition (Oakes et al., 2017, 2019). 2-AG activates TPH-1 expression in ADF neurons, while SER-4 functions in AIB neurons (Oakes et al., 2017). Although 2-AG also promotes DA release from ADE neurons to regulate the locomotion, this pathway is not shown in Figure 1, because of the undefined downstream neurons targets (Oakes et al., 2019).

### 2.3 Feeding

In addition to locomotion, feeding represents another important behavior modulated by 5-HT. Mutation of TPH-1 declines feeding behavior, which is quantified by the rate of pharyngeal pumping (Sze et al., 2000). 5-HT released from ADF neurons alone is sufficient to maintain normal feeding rates, and ADFs show strong calcium responses to bacteria food, whereas such responses are not observed in NSM (Cunningham et al., 2012; Zaslaver et al., 2015; Liu et al., 2019). In contrast to the ADF neurons, NSM neurons respond to environmental cues beyond bacterial food, such as diacetyl or alcohol, and subsequently release 5-HT to promote feeding behavior (Li et al., 2012; Wang et al., 2021).

In the downstream of 5-HT signaling, MC pharyngeal neurons initiate muscle contractions to pump bacterial food from the pharynx into the intestine (Avery and Horvitz, 1989). Loss of MC neuron function leads to sluggish and irregular pumping (McKay et al., 2004; Avery and You, 2012). The GPCR receptors SER-7 is essential for inducing positive E-phase transients in MC neurons and mediating overall acceleration of pumping during exogenous 5-HT-induced feeding (Hobson et al., 2006). ADF neurons release 5-HT to activate SER-7 receptors in both MC and M4 pharyngeal neurons (Hobson et al., 2006; Song and Avery, 2012; Song et al., 2013). SER-5 Mutation in AVJ neurons also results in a defect of 5-HT-induced pumping acceleration (Cunningham et al., 2012; Lemieux and Ashrafi, 2015). Subsequent activation of the AMP-activated protein kinase AAK-2 in AVJ neurons triggers glutamate release, thereby activating downstream signaling pathways (Cunningham et al., 2012; Figure 1, the part with green arrows).

ADF neurons display hypersensitivity when nematodes re-encounter food after a period of starvation (Lemieux et al., 2015; Liu et al., 2019). This hypersensitivity is absent in the TPH-1 mutant, suggesting an increased 5-HT release from ADF in response to food after starvation compared to continuous feeding. This effect is attributed to the deficiency of kynurenine, a tryptophan derivative whose levels drop during food deprivation. Due to the derepression of kynurenine and *N*-methyl-D-aspartate receptor (NMDA-R), downstream neurons release neuropeptide FLP-18 which binds to NPR-5 receptors and activates ADF neuron hypersensitivity (Lemieux et al., 2015). The reporter (*nkat-1p*:GFP) indicates that NKAT-1, a kynurenine-oxoglutarate transaminase for converting kynurenine to kynurenic acid, is expressed in neurons RMDV, RIM, and RID. However, due to uncertain neurons that express NKAT-1 and NMDA-R receptor, the neural circuit map is not presented in the model.

### 2.4 Memory

In the laboratory, the bacterium *Escherichia coli* (*E. coli*) OP50 is commonly used as a food source for nematode feeding. However, in natural environments, bacterial food sources are diverse, including pathogenic strains such as *Pseudomonas aeruginosa* (PA) 14 and *Streptococcus marcessus*, non-pathogenic strains like *E. coli* OP50 and HB101 (Zhang et al., 2005). Odors emitted by pathogenic bacteria elicit aversive learning responses in *C. elegans*. TPH-1 mutants are defect in both aversive and attractive learning, while serotoninergic ADF and NSM neurons exhibit coordinated activity rather than functioning independently in feeding behavior. In this process, ADF neurons play a dominant role, with NSM neurons providing auxiliary support (Zhang et al., 2005). Therefore, only ADF neurons are depicted in the Memory section of Figure 1. The serotoninergic MOD-1 receptor functions in AIZ/AIY neurons (Zhang et al., 2005), thereby, forming a neural pathway from ADF to AIZ/AIY that modulates aversive memory against the pathogenic bacteria (Figure 1, the part with blue arrows).

During PA14-induced aversive learning, the sensory neuron ADF not only releases 5-HT to regulate the downstream neurons, but also receive the regulatory signals from upstream neurons, such as AWB and Amphid wing C cells (AWC) neurons (Qin et al., 2013). Two olfactory neurons AWB and AWC exhibit robust calcium signaling in response to PA14 individually, and modulate the aversive learning to avoid the smell of PA14 bacterium (Ha et al., 2010). EGL-30, the ortholog of vertebrate Gαq protein (Lackner et al., 1999; Bastiani et al., 2003), enhances the activity of ADF neurons and promotes TPH-1 transcription in the aversive training (Qin et al., 2013). The two olfactory neurons AWB and AWC coordinate to regulate ADF neuronal activity for the aversive learning (Figure 1, the part with blue arrows).

AWC olfactory neurons stochastically differentiate into two asymmetric subtypes, AWC^ON^ and AWC^OFF^, both of which are involved in memory storage and retrieval. When worms under adverse conditions (e.g., starvation) are exposed to a specific odor, AWC^ON^ encodes the aversive associative memory between the odor and starvation. After feeding is restored, subsequent odor stimulation alone triggers AWC^OFF^ to promote 5-HT release from NSM/ADF neurons, thereby initiating stress-resistance programs (Eliezer et al., 2019). Remarkably, this associative memory can be transmitted to offspring via epigenetic inheritance, as a result such transgenerational experiential memory improves the survival rate of progeny under adverse conditions (Deshe et al., 2023).

### 2.5 Temperature

When the ambient temperature rises from 20 °C to 26.7 °C, the release of 5-HT elevates pharyngeal pumping rates by 50% (Tatum et al., 2015). The hydroxylase TPH-1 not only elevates the pumping rates but also promotes the expression of heat shock protein 70 (HSP-70) (Tatum et al., 2015). Both ADF and NSM neurons respond to temperature rise, but the ADF sensory neurons exhibit greater efficacy in activating the heat shock transcription factor (HSF1), with a 75% increase in comparison 33% increase observed in NSM neurons (Note: Figure 1 only shows ADF neurons in the Temperature section). AFDs serve as thermosensory neurons that detect temperature changes via guanylyl cyclases GCY-8 and GCY-23 (Clark et al., 2006; Inada et al., 2006; Prahlad et al., 2008; Kuhara et al., 2011). Optogenetic stimulation of AFD neurons elicits an augmentation in pharyngeal pumping, akin to the effect of temperature-induced AFD activation (Tatum et al., 2015). It suggests that AFD neurons facilitate 5-HT release to enhance feeding activity in response to heightened temperatures (Figure 1, the part with red arrows).

### 2.6 Fat storage

Serotonin is an evolutionarily conserved neuromodulator that regulates body fat storage and energy balance. In mammals, pharmacological interventions that elevate 5-HT levels have been shown to reduce body fat (Chan et al., 2013; Oh et al., 2015). Similarly, in *C. elegans*, the application of exogenous 5-HT results in fat loss (Noble et al., 2013). Conversely, the TPH-1 mutation leads to fat accumulation, and this effect is specifically mediated by ADF neurons and is independent of NSM neurons (Noble et al., 2013). Serotoninergic receptor MOD-1 functions in URX neurons to mediate this regulatory pathway, suggesting that 5-HT released from ADF neurons regulates fat loss through MOD-1 receptors in URX neurons (Figure 1, the part with turquoise arrows). This regulatory mechanism requires coordination with OA (Noble et al., 2013), which is another monoamine neurotransmitter widely found in invertebrates (Bauknecht and Jekely, 2017). A more detailed discussion in the section 4.3.

## 3 Neural circuits of OA and TA

Animals release OA and TA in response to adverse environmental conditions, such as food shortage or life-threatening situations (Roeder, 2005; Yang et al., 2015; Selcho and Pauls, 2019). OA and TA are synthesized by TA hydroxylase TBH-1 and tyrosine decarboxylase TDC-1, respectively. Basing on GFP reporter, TDC-1 expression was exclusively observed in two specific neurons, RICs and RIMs, whereas TBH-1 expression was limited to RICs only (Alkema et al., 2005). Consequently, RIC neurons contain relatively low levels of TA because most TA is converted into OA by TBH-1. Due to the functional similarity between OA and TA, the function of RIMs and RICs usually exhibits redundancy; for example, the activity of RIC neurons and RIM neurons both increases during starvation, and exogenous application of either OA or TA reduces feeding rates (Alkema et al., 2005; Suo et al., 2006; Greer et al., 2008; Suo et al., 2009).

**FIGURE 2:**
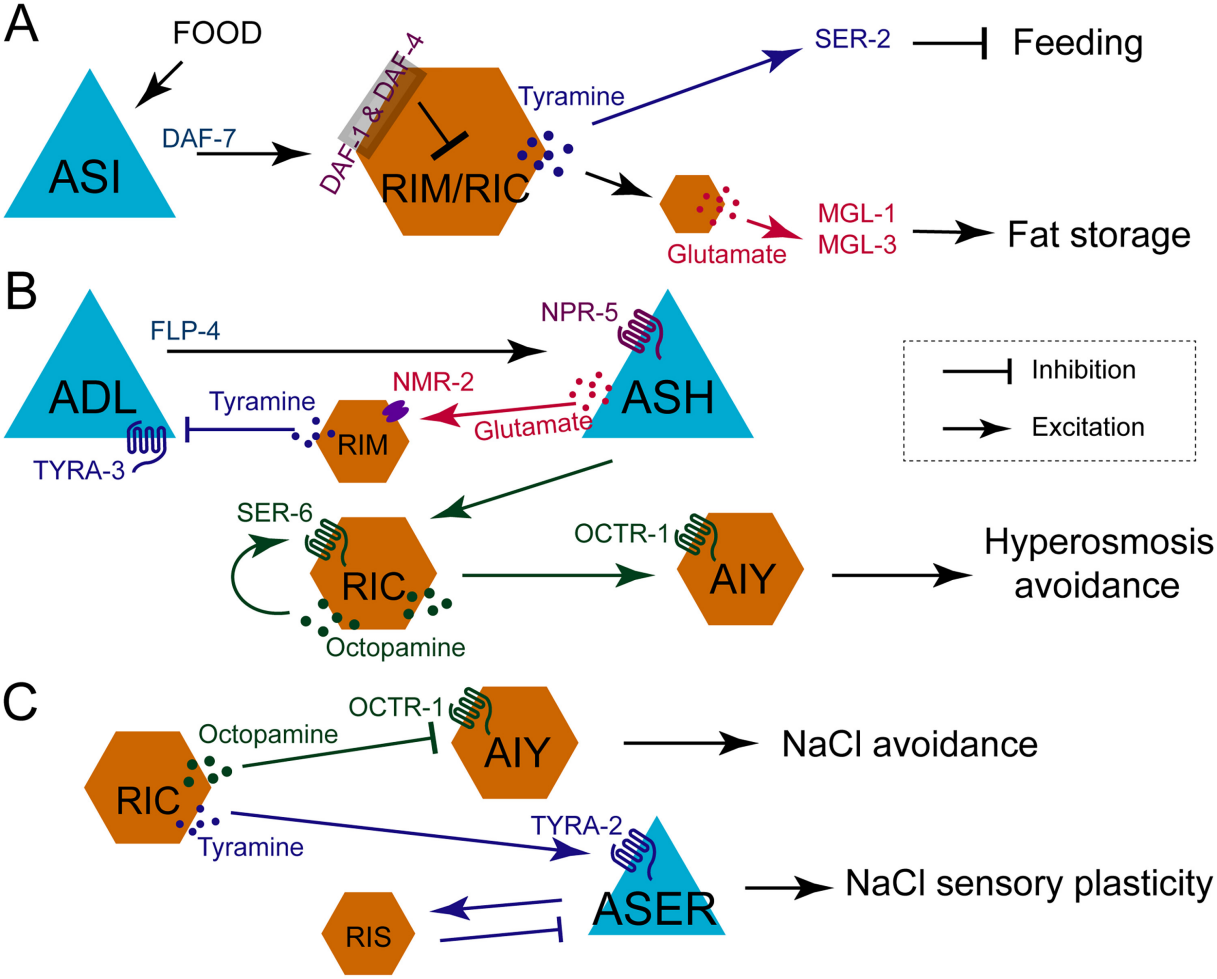
Three-panel scientific diagram depicting neural circuits in *C. elegans* with labeled neurons, signaling pathways, and neurotransmitters. **(A)** Illustrates feeding and fat storage regulation; **(B)** details hyperosmosis avoidance; **(C)** shows circuits for NaCl avoidance and sensory plasticity. Arrows represent excitation, blunt lines indicate inhibition, and specific neurotransmitters and receptors are noted at synapses.

Exogenous OA or TA, as well as mutations in *daf-1* or *daf-7*, all results in a decrease in feeding rates (Greer et al., 2008). DAF-1, a downstream receptor of DAF-7 TGFβ signal (Georgi et al., 1990), functions in both RIM and RIC neurons. Interestingly, in *daf-1*; *tdc-1* double mutants, feeding rates partially recover toward wild-type levels, yet body fat remains elevated—similar to *daf-1* single mutants (Greer et al., 2008). It indicates that the signaling pathways in the downstream of RIM/RIC neurons diverges between feeding control and fat storage regulation, while the upstream neural circuit from ASI neurons to RIM/RIC neurons remains conserved (Greer et al., 2008). This also provides an explanation for why mutations in both DAF-7 and DAF-1 lead to decreased pumping rates and increased body fat, as these outputs are governed by distinct downstream pathways (Figure 2A).

Octopamine and TA also modulate nociceptive responses, such as hyperosmotic avoidance. This behavior involves a feedback circuit comprising ASH, RIM, and ADL neurons. Initially, ASH neurons release glutamate to activate RIM neurons via NMR-2 receptor. TA released from RIM neurons suppress ADL neurons through TYRA-3 receptor. Ultimately, ADL neurons secrete neuropeptide FLP-4 to stimulate ASH neurons via NPR-5 receptor (Liu et al., 2023). In addition, a feedforward circuit (ASH/RIC/AIY) enhances hyperosmotic avoidance: ASH neurons excite RIC via gap junctions, and RIC releases OA to enhance AIY activity through the OCTR-1 receptor (Liu et al., 2023). Notably, RIC neurons also release OA to potentiate their own activity via the SER-6 receptor, likely amplifying the signal to ensure effective escape from hyperosmotic stress (Figure 2B; Liu et al., 2023).

Learning and memory represent fundamental survival mechanisms in animals. A neural circuit comprising ASER, RIC, RIS, and AIY governs short-term salt chemotactic learning in nematodes (Figure 2C). After brief salt adaptation, calcium activity decreases in RIC and ASER neurons but increases in AIY. Salt stimulation suppresses RIC activity, thereby inhibiting the downstream ASER neuron and alleviating the inhibition of AIY neurons via the TA/TYRA-2 and OA/OCTR-1 signaling, respectively (Wang et al., 2025). Notably, the ASER sensory neuron not only receives tyraminergic input from RIC neuron but also integrates negative feedback from RIS neuron. AIY and ASE neurons appear to perform distinct functional roles: AIY regulates salt avoidance, whereas ASER detects salt and retains taste memory (Wang et al., 2025).

Many neural circuits involving RIM and RIC neurons depend on the synergistic interaction of DA and 5-HT signaling, thus these circuits have been incorporated into different contexts, such as Locomotion depicted in the sections 2.2, 4.4 and 4.5; Lifespan in 2.1; Feeding in 2.3, 4.1 and 4.6; and Fat in 4.3.

## 4 Interaction of biogenic amines in neural circuits

### 4.1 Food stimuli

During starvation, OA released from RIC neurons induces gene expression in SIA neurons by cAMP-response element binding protein (CREB). CREB activation is facilitated by the octopaminergic receptor SER-3 and the Gαq protein EGL-30, and inhibited by Gαo GOA-1 (Miller and Rand, 2000; Suo et al., 2006). Mutations in CAT-2, the rate-limiting enzyme for DA synthesis (Lints and Emmons, 1999), upregulate CREB expression.

**FIGURE 3:**
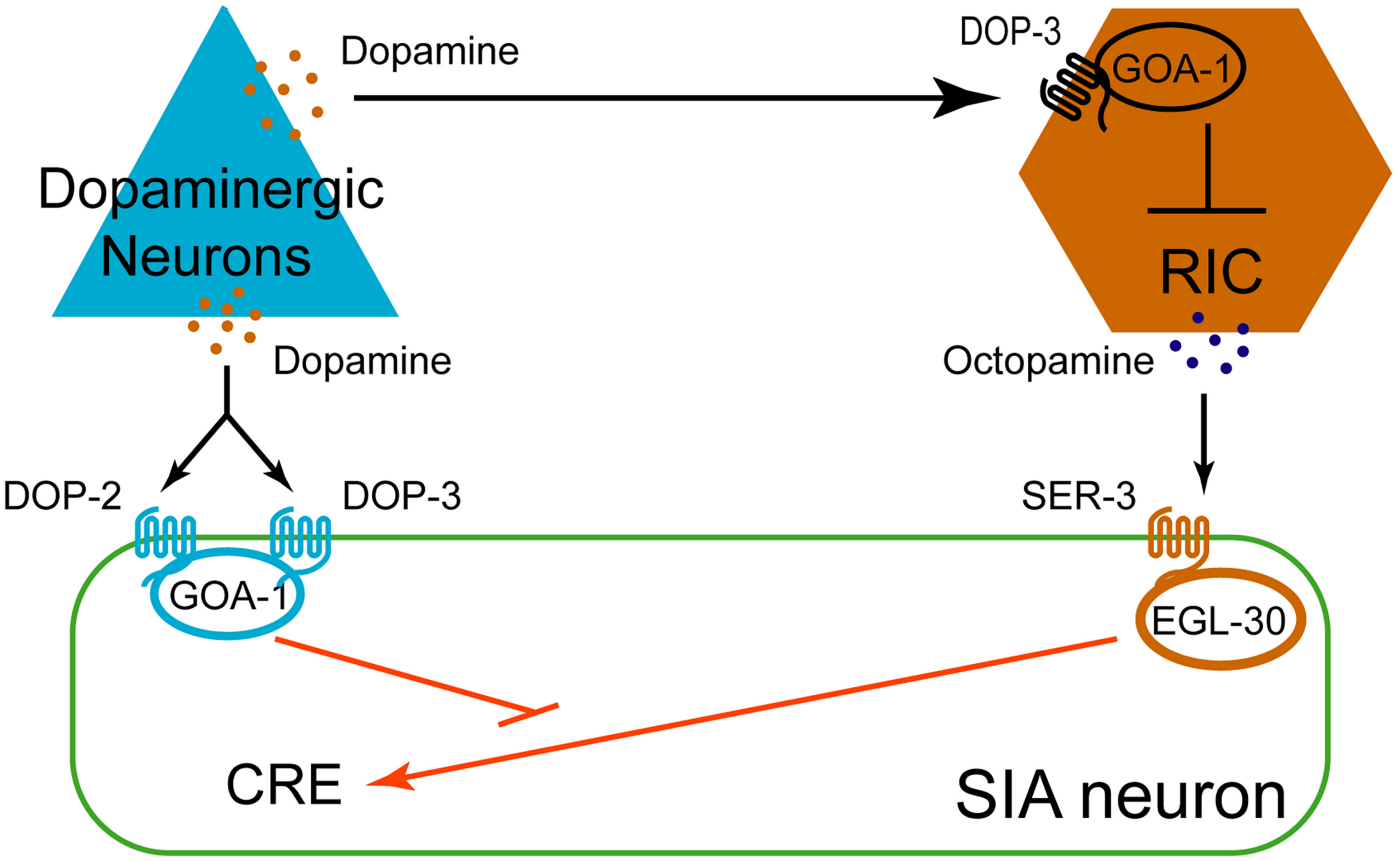
Diagram illustrating dopaminergic neurons releasing DA, signaling via DOP-2 and DOP-3 receptors to the SIA neuron expressing GOA-1, and to an RIC neuron expressing GOA-1, which inhibits OA release; OA acts on the SIA neuron via the SER-3 receptor and affects EGL-30 signaling.

Dopamine downregulates CREB expression through two receptors, DOP-2 and DOP-3 (Suo et al., 2009). Because dopaminergic neurons respond to both food source and mechanical stimulation, it is necessary to determine whether DA release is triggered by the food itself or by the mechanical force associated with bacterial feeding. Sephadex beads, a small bead that mimics the shear forces generated by food, can elicit DA release and subsequently reduce CREB expression (Suo et al., 2009). The DOP-2 in SIA neurons responds specifically to food stimulation but not to Sephadex. In contrast, DOP-3 in both SIA and RIC neurons, responds to mechanical stimulation generated by Sephadex beads and effectively inhibits CREB expression (Suo et al., 2009). These findings suggest that dopaminergic neurons, together with RIC and SIA neurons, constitute a neural regulatory circuit, as illustrated in Figure 3.

### 4.2 A flip-flop circuit for feeding regulation

**FIGURE 4:**
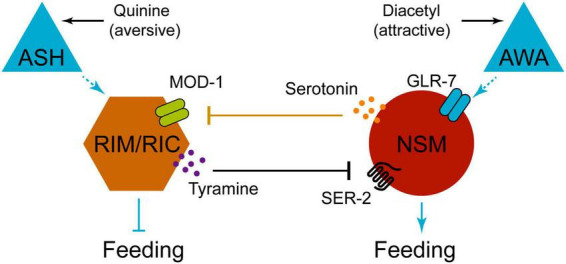
Diagram illustrating neuron interactions influencing feeding behavior. ASH neuron responds to aversive stimulus quinine, AWA neuron to attractive diacetyl. RIM/RIC neuron signals to NSM via tyramine and SER-2, while NSM signals back via serotonin and MOD-1 Feedings are indicated downstream of both pathways.

The reciprocal regulation of serotoninergic neurons and tyraminergic neurons forms an intriguing neural circuit, resembling a bistable flip-flop switch, which integrates two opposing sensory signals to modulate pumping rates (Li et al., 2012). Two sensory neurons ASH and AWA respond to the aversive stimulus quinine and the attractive stimulus diacetyl, respectively. In the downstream neurons, TDC-1 mutations in RIM/RIC neurons abolish the inhibitory effect of quinine on pumping behavior; similarly, TPH-1 mutations in NSM neurons eliminate the diacetyl-induced enhancement of pumping (Li et al., 2012). Intriguingly, 5-HT from NSMs act on receptor MOD-1 in RIM/RIC neurons, and TA from RIM/RIC neurons act on receptor SER-2 in NSM neurons (Li et al., 2012). Thus, this flip-flop circuit that integrates two contradictory sensory inputs generates bistable bioamines output to regulate feeding behavior (Figure 4).

### 4.3 Fat metabolism

**FIGURE 5:**
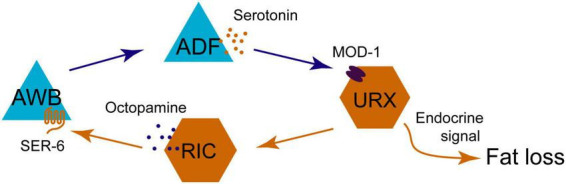
Diagram illustrating neural and hormonal signaling pathways for fat loss, showing AWB, ADF, RIC, and URX cells with arrows representing serotonin and octopamine release, MOD-1 and SER-6 signaling, and an endocrine signal leading to fat loss.

Both exogenous 5-HT and OA reduce intestinal fat of *C. elegans*. Notably, the combined administration of low doses (2.5 mM) 5-HT and OA leads to more significant fat loss compared to a double dose (5 mM) of either 5-HT or OA alone (Noble et al., 2013). TBH-1 mutation that lacks OA does not significantly alter body fat, but it results in a significant reduction of exogenous 5-HT-induced fat loss. Similarly, TPH-1 mutation also significantly suppresses exogenous OA-induced fat loss (Noble et al., 2013). It suggests that 5-HT and OA are interdependent roles in regulating fat loss. Mutations of 5-HT receptor MOD-1 or OA receptor SER-6 each partially reduce fat loss induced by exogenous 5-HT or OA, while the double mutation *mod-1*; *ser-6* results in no fat loss regardless of both exogenous additions (Noble et al., 2013). In this neural circuit, the receptor MOD-1 and SER-6 function in URX neurons and AWB neurons, respectively (Noble et al., 2013). Considering the regulatory roles of URX and RIC neurons in 5-HT-induced fat loss and the direct synaptic connection between them, it is possible that there exists a neural feedback circuit from URX and RIC to ADF neurons (Figure 5).

### 4.4 A mutual inhibitory circuit for movement regulation

**FIGURE 6:**
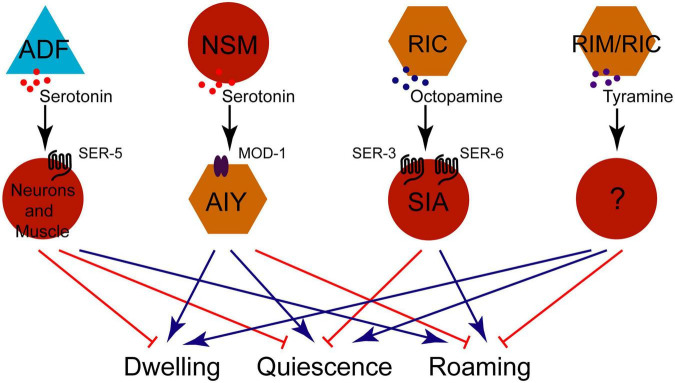
Diagram illustrating neurotransmitter signaling pathways in *C. elegans* affecting behavior. Shapes represent neuron types, arrows indicate neurotransmitter release and receptor interactions, leading to dwelling, quiescence, or roaming behaviors.

In *C. elegans*, the nervous system regulates three distinct behavioral states: roaming, dwelling, and quiescence. Roaming and dwelling can be distinguished by the parameters such as movement velocity and body wave characteristics. Quiescence is characterized by a complete cessation of movement (Ghosh and Emmons, 2008; Churgin et al., 2017; McCloskey et al., 2017). The presence of bacterial food leads to an increase in quiescence and dwelling, and a decrease in roaming. TPH-1 mutations, which lack serotonin (5-HT), exhibit reduced quiescence and increased roaming (Churgin et al., 2017). Interestingly, unlike their previously described cooperative roles in feeding and lifespan, 5-HT released from ADF and NSM neurons exerts opposing effects on these behavioral states (Figure 6). ADF neurons release 5-HT to promote roaming by SER-5, while NSM neurons release 5-HT to promote dwelling and quiescence by MOD-1 in AIY neurons (Churgin et al., 2017). The previous description has clarified that TA and OA manifest analogous functions (Alkema et al., 2005). In this behavioral regulation, they possess distinct roles. OA binding to SER-3 and SER-6 receptors on SIA neurons reduces quiescence and increases roaming (Churgin et al., 2017). Conversely, TA promotes quiescence and suppresses roaming (Figure 6). These questions merits further investigation: Why does 5-HT released from different neurons exhibit completely opposite regulatory functions? What mechanisms underlie the functional antagonism between OA and TA?

### 4.5 A seesaw model for avoidance of cu^2+^

**FIGURE 7:**
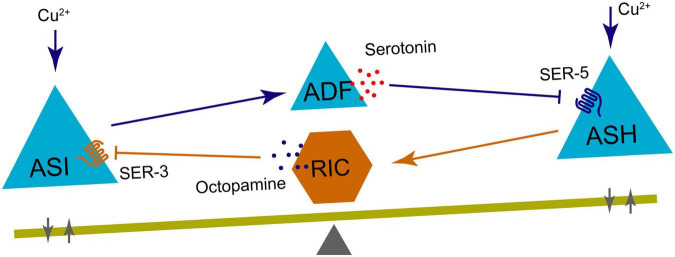
Diagram showing neural interactions involved in Cu^2 +^ response where ASI and ASH neurons are activated by copper ions, ADF neuron releases serotonin to ASH via SER-5, and RIC neuron releases octopamine to ASI via SER-3 and to itself. A balance beam at the bottom with a fulcrum symbolizes balance between these pathways.

A mutual inhibitory neural circuit regulates the avoidance behavior of Cu^2+^. Specifically, blocking ASH neurons with TeTx resulted in increased calcium signaling in ASI neurons in response to Cu^2+^, while blocking ASI neurons led to heightened calcium signaling in ASH neurons (Guo et al., 2015). These findings suggest that cross-inhibitory neural circuits between the sensory neurons ASH and ASI regulate adaptive avoidance responses to Cu^2+^. Furthermore, RIC neurons and ADF neurons, like mediators, mediate this cross-inhibition (Figure 7). Additionally, a subtle feature emerges in the interaction between these neurons: neural excitation from ASIs to ADFs is not able to alter calcium signaling intensity of ADF neurons, but accelerate the Cu^2+^ response time of ADF neurons (Guo et al., 2015).

The question then arises: Which of the two neurons, ASH or ASI, sent out instruction to guide animal behavior? First, let us analyze the primary functions of ASH and ASI neurons. ASH neurons function as polymodal nociceptors, responding to noxious stimuli, such as hypertonic pressure (Bargmann et al., 1990), nose touch (Kaplan and Horvitz, 1993; Hilliard et al., 2005), volatile repellent chemicals (de Bono and Maricq, 2005), heavy metals (Hilliard et al., 2005), high salts (Chatzigeorgiou et al., 2013) and quinine alkaloids (Hilliard et al., 2004). In contrast, ASI neurons regulate animal behavior by coordinating or counterbalancing other neural circuits, such as promoting local search behavior triggered by AWC and ASK neurons (Gray et al., 2005), coordinating temperature memory behavior by AFD neurons (Beverly et al., 2011), and antagonizing ASJs and ASKs promotion of dauer entry (White and Jorgensen, 2012). Thus, it can be inferred that ASIs may function merely as regulators to balance animals’ nociception. The second question, Whether this mechanism could be linked to animal hesitancy in the face of adversity? The delicate balance of mutual inhibition between ASIs and ASHs leads animals to exhibit hesitation in choosing an escape direction during injury from the heavy metal ion Cu^2+^. For instance, in a four-quadrant choice experiment, some worms were observed still crawling in the Cu^2+^ region even after a duration of 30 min (Guo et al., 2015). This might highlight the disparity between biological neural thinking systems and computer programs, as the outcomes produced by the latter are easily envisioned. Therefore, the analysis of behavioral experiments heavily relies on the outcomes of Gaussian distribution.

### 4.6 A disexcitation mechanism for feeding regulation

**FIGURE 8:**
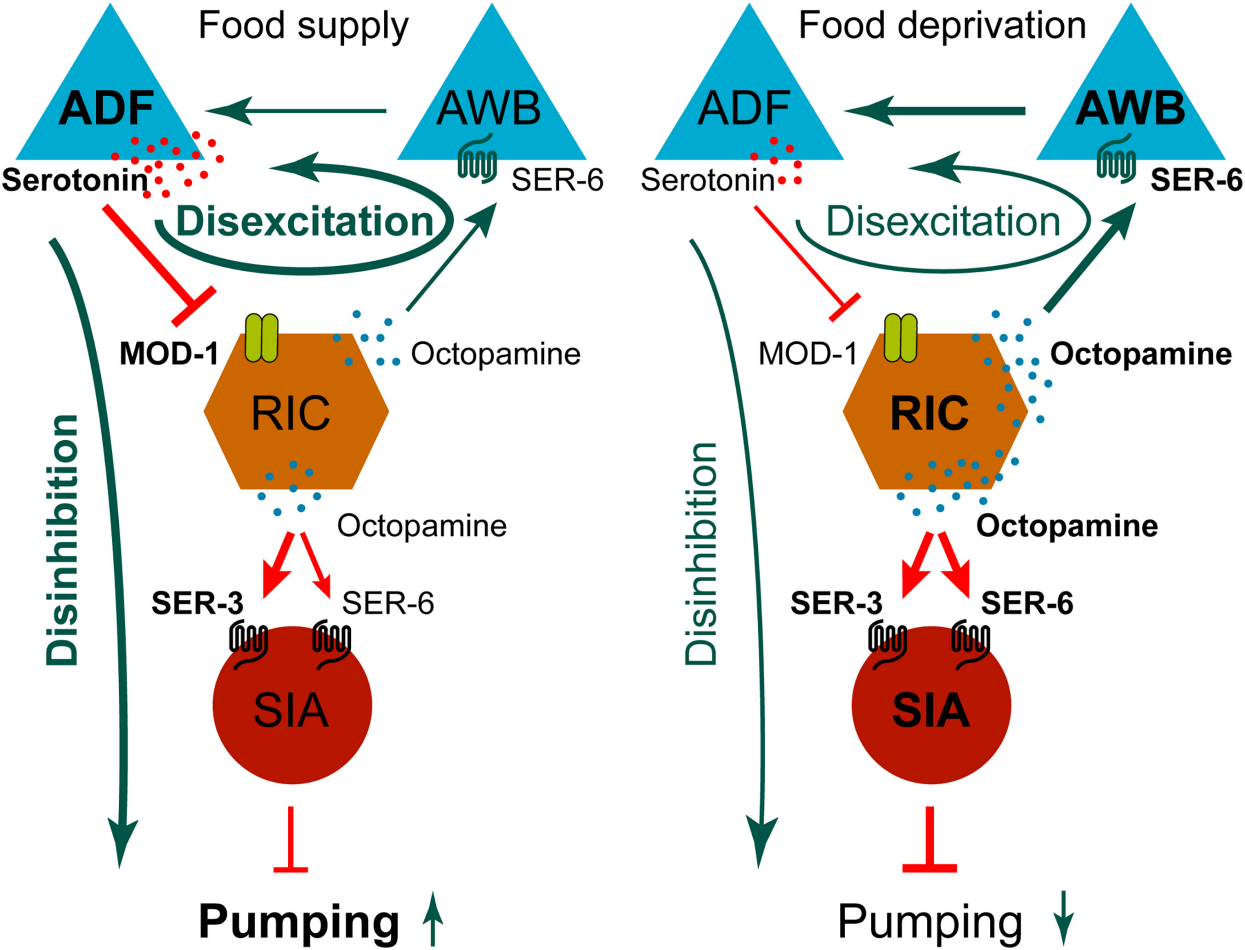
Diagram comparing neural circuit activity in food supply versus food deprivation conditions, showing triangles labeled ADF and AWB, hexagon labeled RIC, and circle labeled SIA. Arrows indicate serotonin and octopamine signaling, disexcitation, disinhibition, with SER-3, SER-6, and MOD-1 receptors. Food supply and deprivation result in different signaling pathways influencing SIA-mediated pumping.

The pumping behavior has long attracted significant research interest. As previously described, ADF neurons promote 5-HT production in response to bacterial food. In the downstream of ADF neurons, 5-HT inhibits RIC neurons by a chloride channel receptor MOD-1 in well-fed. OA from RIC neurons acts on two difference receptors: SER-3/SER-6 in SIA neurons and SER-6 in AWB neurons in starvation. A feedforward neural circuit is composed of three neurons: ADF, RIC and SIA with a simple mechanism, disinhibition, which lead to pumping increase (Liu et al., 2019). And an ADF → RIC → AWB → ADF neural feedback circuit regulates pumping rates with a disexcitation mechanism (Figure 8).

Notably, the upstream pathway that ADFs release 5-HT to inhibit RICs through MOD-1 is shared by both circuits. This means that disinhibition and disexcitation occur simultaneously during well-fed, and simultaneously decay with gradual decline of food concentration. Consequently, maintaining homeostasis of ADF activity, especially the level of 5-HT production, plays a crucial role in regulating the pumping behavior. An important question arises: Why the excitatory pathway from RIC neurons, AWB neurons to ADF neurons do not increase pumping rates when OA levels rise during starvation? The reason lies in the saturation of AWB neuron activity stimulated by OA signals from RIC, as observed through dosage test (Liu et al., 2019).

This regulatory mechanism has important implications for survival in fluctuating environments. When food is abundant, disinhibition of the neural feedforward circuit ADF → RIC → SIA increases pumping rates to acquire more food resources. Under food deprivation, reduced 5-HT production attenuates disexcitation in the ADF/RIC/AWB/ADF feedback circuit, thereby preserving a baseline level of pumping. This regulatory arrangement serves two main purposes. First, it prevents an excessive decline in pumping [blocking AWB neurons or mutating SER-6 during starvation reduces pumping by ∼60% or more (Liu et al., 2019)], allowing feeding rates to rebound rapidly when food is re-encountered. Secondly, it avoids excessive high level of 5-HT production during starvation through preventing excessive serotonergic activation caused by saturating activation of AWBs stimulated by OA (Liu et al., 2019).

## 5 Conclusion and outlook

Although only four biogenic amines have been identified in *C. elegans*, these signaling molecules are capable of modulating a wide range of behavioral responses by diverse regulatory mechanisms. The complex spatial instructions of behavior originate from the interaction of different neurons in neural circuits mediated by multiple neurotransmitters and receptors, and are implemented through excitation, inhibition, feedforward, and feedback. These behavioral and metabolic regulations involve multi-level neurophysiological mechanisms of neural signaling.

If neurotransmitters are conceptualized as behavioral instructions encoded by neurons, a simplified view would suggest that the same neurotransmitters always mediates the same function, while different types of neurotransmitters are mutually antagonistic. For instance, 5-HT released from both ADF and NSM neurons promotes feeding behavior in response to different food stimuli (Liu et al., 2019), and increased levels of OA or TA reduce feeding rates (Li et al., 2012). In Cu^2+^ avoidance, ASH and ASI neurons reciprocally inhibit via an OA-5-HT “seesaw” mechanism (Guo et al., 2015). However, the mutually antagonistic neuromodulators, 5-HT and OA, exhibit cooperative functions in lipid metabolism (Noble et al., 2013). More intricately, the same neurotransmitter can have opposite regulatory effects on the same behavior. For example, OA exhibits both excitatory and inhibitory functions in feeding behavior (Liu et al., 2019), and 5-HT has opposite functions because it originates from different neurons (Figure 6; Churgin et al., 2017). Additionally, there are numerous astonishing tricks. Such as, ASI neurons do not directly excite ADF neurons but accelerate the response speed to modulate the copper avoidance behavior (Guo et al., 2015). And AWB neurons set a threshold to prevent excessive hypersensitivity in response to OA signals (Liu et al., 2019).

Neural circuits regulate fundamental desires and behaviors of animals by integrating the complex neurotransmitter signals, such as the drive to eat when experiencing hunger. Evidences show that feeding behavior decreases when the 5-HT synthase TPH-1 is loss of function, and 5-HT is released hypersensitively in response to food odors when the animal is hungry (Liu et al., 2019). It suggests that 5-HT regulates the feeding instinct of the animals, while eating also induces 5-HT release, and this release seems to elicit a sense of satisfaction. Another form of mutual inhibition within neural circuits can explain indecision in psychological phenomena, exemplified by the seesaw response of ASH and ASI neurons to copper ions (Guo et al., 2015). Furthermore, a model of the flip-flop circuit between RIM/RIC and NSM neurons in response to aversive and attractive foods (Li et al., 2012), explains the significance of first impressions in determining whether people accept or reject a food source. More notably, a dopamine-mediated model of economic value evaluation demonstrated that *C. elegans* assigns a lower value to an inefficient food compared to normal food. Consequently, when the two types of food are mixed in varying concentrations, *C. elegans* weighs the costs and benefits and select the appropriate one (Millet et al., 2025). More interestingly, *C. elegans* achieves transgenerational memory through epigenetic marks, passing on survival experiences to the offspring and enabling them to better resist environmental adversities (Deshe et al., 2023).

In summary, neurotransmitters play a critical role in regulating unconscious behaviors. However, the precise mechanisms through which they modulate subjective consciousness and complex behaviors remain to be fully elucidated. Given that the brain is an extremely complex system, understanding its functional architecture fundamentally depends on deciphering the connectivity and coordination among neural circuits. Some scholars even expect to comprehensively analyze the regulation and integration of information flow through whole-brain neural perspective (Calhoun et al., 2014; Randi et al., 2023; Munn et al., 2024). The research in this field offers direct insights into the neural substrates underlying specific cognitive functions and behavioral outputs. Importantly, many neuropsychiatric disorders do not arise from widespread brain damage but rather from dysfunctions or disruptions in specific neural circuits. Therefore, investigating neural circuits represents a pivotal approach for uncovering the pathophysiological mechanisms of these diseases.
